# An Anti-Vivisectionist Manifesto

**Published:** 1909-01-23

**Authors:** 


### The Hospital

A Journal of

The Medical Sciences and Hospital Administration.

New Series. No. 99, Vol. IV. [No. 1171, Vol. XLV.] SATURDAY, JANUARY 23, 1909.

### AN ANTI-VIVISECTIONIST MANIFESTO.

A good many, and perhaps all, holders of licenses for vivisection have lately been indulged with a communication of so singular a character that we are tempted to share it with our readers. It supplies an illuminating commentary upon the complex psychology of the anti-vivisectionist mind and is a monument of sincerity and self-opinion. It emanates from an individual called M. Cowan, whose modesty precludes any further revelation of identity. We thus remain in ignorance both of the sex and of the residence of this zoophilist, and must be content with respecting the reserve which prompts such doing of good in secret. The communication is a manifesto printed upon foolscap, and begins as follows: "Some little time ago, in the coffee-room of a London hotel, I chanced to hear one of the party at a table close by narrate how he knew a person who was in the habit of praying from time to time for the death of one of our leading vivisectors: he said that always the man indicated had died. I tried to trace the speaker, but, as time had elapsed, did not succeed in doing so; I then thought, as I myself know of the efficiency of prayer, it would be well to try if this were actually so. I thought first of experimenting on Dr. Starling, but it seemed to me unfair to give such a stab in the dark without first letting it be known what was intended. It seemed also almost cruel, without knowing any of the surrounding circumstances, to select at random one from the large number of distinguished scientists on the medical lists. It was, therefore, finally decided to make earnest prayer, giving much thought to the subject, that the Almighty, if the prayer were in accord with His will, would promptly remove the man most likely to cause future suffering to innocent subjects by his experiments. About a fortnight later one of our most distinguished medical scientists dropped, and the newspapers were lamenting the loss to science of this vivisector, and the discoveries he was just about to make."

With this exordium the writer proceeds, by dint of scriptural quotation, to demonstrate the Divine presence "in the laboratory beside the operating trough," whereby, curiously, the animal experimented upon often obtains an insensibility to agony which seems unaccountable to the operator. Denouncing the title of any Royal Commission to lay down laws for the conduct of a practice inherently immoral, the writer warns us that "the prayer of the child of God reaches further, is more searching in its effects, than any enactment of the law can be. Not only does it enter the vivisecting room, embracing all operations there, it is with the patients in our hospitals and by the bedside of the poorest sufferer, and it will make its way into the garret or concealed chamber where the student, eager for knowledge and advancement, illegally tortures his helpless victim from day to day." The latter part of this paragraph has a familiar ring and is in the best style of the more ordinary anti-vivisector. But thereafter the document becomes rambling. "For many years," says the writer, "in fact since 1877, the subject of vivisection has been greatly and distressingly in my thoughts." Decidedly it has, and the most hardened vivisector will not forbear to wish him (or her) well rid of it, for clearly it is doing harm. The remainder of the manifesto does not lend itself to comment in a public print. It is a remarkable, and not a little pathetic, example of the singular mixture of humility and conceit which pervades a good many fervently religious minds: of the humility which is profoundly conscious of unworthiness, of the conceit which nevertheless supports the hope of giving suggestions for conduct to the Creator.

We should not have held it worth while to give the mysterious M. Cowan this publicity were it not that we have in his excursion the *reductio ad absurdum* of the anti-vivisectionist mode of thought. Here we have the ready acceptance of hearsay when it jumps with preconceptions (for who that had witnessed animal experiments would speak of the "operating trough"?); the inexplicable confusion of mind which credits the Divine presence with the dissipation of pain in animals subjected to vivisection, yet makes the Creator so eclectic in His favours that the prayers of all true believers are invoked to remind Him of His duties towards vivisectors; the rioting imagination which finds no difficulty in conjuring up a remorseless student furtively carrying out his hateful experiments in a garret. The peculiar faculty here illustrated is of the essence of the anti-vivisectionist crusader, for let him but cultivate a judicial mind and he must recant. Put crudely, as they are in the pamphlet before us, the considerations advanced are so laughable as to be harmless. But the same items, properly sauced by more experienced and less fanatical cooks, appear in every anti-vivisectionist *menu*, and are the origin of much tiresome mental indigestion to a host of impressionable and sincere but ignorant people. It is not every day that one is introduced to the professed humanitarian who calmly contemplates "experimenting on Dr. Starling" by prevailing upon Providence to cut short his physiological studies in the drastic fashion suggested, but an analogous lack of charity and a similar assumption of monopoly in right-thinking is a familiar feature of anti-vivisectionist polemics. If it were possible that the confirmed fanatics who flaunt the flag of Mr. Stephen Coleridge and his allies might be made amenable to reason we can imagine nothing more likely to produce that happy consummation than a perusal of the Cowan manifesto. *Similia similibus curantur.* But we fear they are past praying for.

